# Cable bacteria delay euxinia and modulate phosphorus release in coastal hypoxic systems

**DOI:** 10.1098/rsos.231991

**Published:** 2024-04-17

**Authors:** Laurine D. W. Burdorf, Sebastiaan J. van de Velde, Silvia Hidalgo-Martinez, Filip J. R. Meysman

**Affiliations:** ^1^ Geobiology Research Group, Department of Biology, University of Antwerp, Antwerp, Belgium; ^2^ Department of Biotechnology, Delft University of Technology, Delft, The Netherlands

**Keywords:** cable bacteria, iron cycling, phosphorus, hypoxia, euxinia

## Abstract

Cable bacteria are long, filamentous bacteria with a unique metabolism involving centimetre-scale electron transport. They are widespread in the sediment of seasonally hypoxic systems and their metabolic activity stimulates the dissolution of iron sulfides (FeS), releasing large quantities of ferrous iron (Fe^2+^) into the pore water. Upon contact with oxygen, Fe^2+^ oxidation forms a layer of iron(oxyhydr)oxides (FeO_x_), which in its turn can oxidize free sulfide (H_2_S) and trap phosphorus (P) diffusing upward. The metabolism of cable bacteria could thus prevent the release of H_2_S from the sediment and reduce the risk of euxinia, while at the same time modulating P release over seasonal timescales. However, experimental support for this so-called ‘iron firewall hypothesis’ is scarce. Here, we collected natural sediment in a seasonally hypoxic basin in three different seasons. Undisturbed sediment cores were incubated under anoxic conditions and the effluxes of H_2_S, dissolved iron (dFe) and phosphate (PO_4_^3−^) were monitored for up to 140 days. Cores with recent cable bacterial activity revealed a high stock of sedimentary FeO_x_, which delayed the efflux of H_2_S for up to 102 days. Our results demonstrate that the iron firewall mechanism could exert an important control on the prevalence of euxinia and regulate the P release in coastal oceans.

## 1. Introduction

Oxygen concentrations in coastal waters are decreasing as a result of global change (IPCC report [1]). An increased nutrient run-off from land in combination with warming waters leads to an increase in the spatial extent, temporal extent and frequency of bottom-water oxygen depletion [2–4]. The development of bottom-water hypoxia ([O_2_] < 63 µmol l^−1^) is typically a seasonal phenomenon linked to the stratification of the water column in spring and summer, which reduces the replenishment of the bottom water with oxygen-rich surface water [3].

Bottom-water hypoxia substantially impacts the seafloor ecosystem functions related to macrofauna such as bioirrigation and bioturbation (e.g. [5,6]). This impact can be particularly aggravated when anoxia develops and eventually free sulfide escapes from the sediment and accumulates at the bottom of the water, a condition referred to as euxinia. Under fully oxygenated bottom waters, free sulfide (H_2_S) is efficiently oxidized in the top layer of the sediment and hence, it does not escape to the overlying water. However, when bottom waters become anoxic, the sediment releases H_2_S and euxinia develops. The latter condition can have important ecological and economic consequences, as H_2_S is highly toxic to fauna [7]. While seasonal hypoxia is observed more frequently and for longer time periods in coastal waters [8], the reports of euxinia are relatively rare (except for permanently stratified systems such as the Baltic and Black Sea). So why is euxinia not more prominent in coastal environments? And will the prevalence of euxinia increase with the ongoing global change?

A study by Seitaj *et al*. [9] proposes that the relative infrequency of euxinia in coastal bottom waters can partly be explained by an ‘iron firewall’ mechanism. This mechanism implies strong seasonal switches in the iron and sulfur geochemistry of the sediment, which are induced by the metabolic activity of a specific type of sulfide-oxidizing bacteria, called cable bacteria [9]. Cable bacteria form long filaments that can spatially separate two redox half-reactions of aerobic sulfide oxidation by inducing electric currents over centimetre-scale distances [10–13]. The bottom cells of the cable bacterium filaments oxidize free sulfide in deeper sediment layers and then transport the electrons from cell to cell to the top cells, which reduce the oxygen near the sediment–water interface. This spatial separation of two redox half-reactions also implies a spatial separation of proton production and proton consumption in the sediment. Electrogenic sulfur oxidation (e-SO_x_) causes the acidification of the deeper anoxic zone, while oxygen reduction entails an alkalization of the shallow oxic zone, thus leading to significant pH excursions with depth [12,14,15]. The acidification of deeper sediment layers (>2 pH units, e.g. [16,17]) also leads to the dissolution of particulate iron monosulfides (FeS), which releases ferrous iron (Fe^2+^) and free sulfide (H_2_S) into the pore water [15,18]. The free sulfide is immediately scavenged by cable bacteria, which have a high affinity for free sulfide and use it as an electron donor [14]. The ferrous iron accumulates in the pore water and diffuses to the top layer of the sediment, where iron(oxyhydr)oxides (FeO_x_) are formed upon contact with oxygen [9,18,19]. FeO_x_ accumulation near the sediment surface also occurs without the activity of cable bacteria, through the sedimentation of FeO_x_ from the water column [20,21]. Still, the oxidation of FeS by cable bacteria has the potential to substantially increase this FeO_x_ pool within the surface sediment [9].

A five-year survey of a seasonally hypoxic lake (Lake Grevelingen, The Netherlands) uncovered that cable bacteria are abundant and active in the sediment in winter and spring, prior to the onset of hypoxic conditions. In this period, the cable bacterial activity correlated with the formation of a large enrichment of FeO_x_ in the top sediment layer. In late spring and summer, this FeO_x_ reservoir gradually disappeared, likely by reduction with H_2_S diffusing from below, thus preventing an efflux of H_2_S from the sediment. Seitaj *et al*. hypothesized that by generating this ‘iron firewall’ before the onset of anoxia, cable bacteria could delay or even avoid the occurrence of euxinia in seasonally hypoxic basins [9].

The formation and dissolution of an FeO_x_ layer at the sediment surface also affect the cycling of phosphorus (P) [22,23]. The degradation of organic matter and reduction of FeO_x_ onto which inorganic phosphate is adsorbed in the sediment provide the source of P to the pore water [24]. FeO_x_ strongly bind to P and so the formation of FeO_x_ can efficiently trap P released by organic matter mineralization [23]. Consequently, the large pool of FeOx created by cable bacteria in spring has the potential to retain P in the sediment [25]. Field studies from Lake Grevelingen have indeed demonstrated a zero efflux of P from the sediment in the spring period, when high rates of FeO_x_ formation occur, stimulated by the e-SOx activity of cable bacteria [25].

Cable bacteria seem to thrive particularly well in seasonally hypoxic basins [9,16,17,26–28], so the iron firewall mechanism could be potentially widespread in stratified coastal basins. To date, however, no experimental verification has been provided that cable bacterial activity can indeed prevent H_2_S effluxes from sediments and hence delay euxinia. As a result, various aspects remain unclear such as How long can the iron firewall mechanism delay euxinia? How does it modulate P effluxes from the sediment? And does the firewall strength change when the sediment has been exposed for a longer time to hypoxic conditions? Here, we report on a detailed experimental investigation of the iron firewall mechanism and its impact on sulfur and phosphorus effluxes from the coastal sediment. To this end, intact sediment cores were collected from Lake Grevelingen at key time points within the seasonal hypoxia cycle and these cores were subsequently exposed to anoxia during laboratory sediment incubations. Fluxes were documented at weekly resolutions, to verify the timing and strength of the iron firewall and to examine how the sediment geochemistry evolves ‘en route’ towards the state of euxinia.

## 2. Material and methods

### 2.1. Site description and field sampling

**Figure 1.**
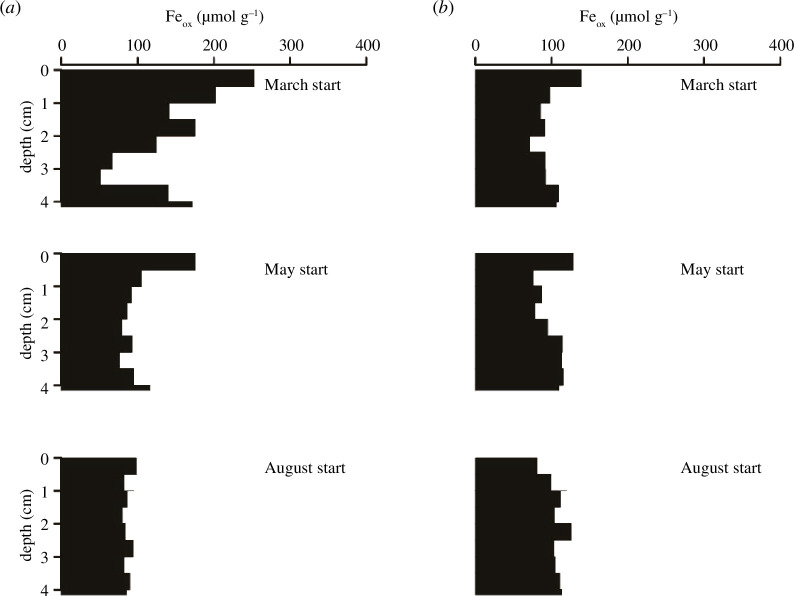
(*a*) Location of Lake Grevelingen, a saline seasonal hypoxic lake in The Netherlands. (*b*) Sampling station ‘S1’ is located in one of the deeper gullies in the south-west part of the basin. (*c*) Oxygen dynamics in Lake Grevelingen based on the data of the monitoring program by Rijkswaterstaat (Dutch Ministry of Infrastructure; https://waterberichtgeving.rws.nl/monitoring/tso-metingen/grevelingenmeer) between 1995 and 2011. Black dots are the median monthly concentrations, red dots are ±25% quantiles. In 2015, cores were taken at three time points (indicated by the arrows) and the *in situ* oxygen concentrations are plotted as green dots.

Lake Grevelingen is a coastal saltwater reservoir located in the Rhine–Meuse–Scheldt delta area in The Netherlands (figure 1*a,b*), which originated after an estuarine branch was closed off from the North Sea by a dam. Oxygen depletion is a yearly recurring feature in deeper bottom waters (>15 m, figure 1*c* [29]). The sediments of these deeper sections are fine-grained (median grain size: 16 µm), organic-rich (3.1 weight %C), have a high CaCO_3_ content (22% by weight) and a sediment accumulation rate of ~1.6 g cm^−2^ yr^−1^ [17,18]. For the present study, we retrieved sediment cores from station ‘S1’ in the Den Osse basin (51° 44′ 46.3″ N 3° 52′ 45.1″ E, a water depth of 23 m; figure 1*b*).

Five sediment cores (UWITEC gravity corer: cores: 60 cm length and inner diameter: 60 mm) were retrieved at three distinct time points in the seasonal hypoxia cycle in 2015 (March: fully oxic bottom water; May: start of the oxygen decline; August: end of the hypoxic period; figure 1*c*). We recorded microsensor depth profiles ship-board, at *in situ* temperatures and within 6 h of sediment sampling. All sediment cores were subsequently transferred to an onshore laboratory and two cores were immediately sectioned for the retrieval of the pore water and solid-phase samples, while three other cores were used for long-term sediment incubations. A vertical depth profile of oxygen in the water column was recorded by a conductivity temperature and depth (CTD) profiling instrument fitted with an O_2_ optode (YSI6600). The bottom water was collected using a 12 l NISKIN bottle and the O_2_ concentration was determined by Winkler titration in three replicate samples.

### 2.2. Microsensor profiling

The microsensor depth profiles of O_2_, pH and H_2_S (*n* = 3–4 replicates in each core) were recorded using microelectrodes (Unisense A.S. Denmark, tip sizes pH: 200 μm, H_2_S: 100 μm and O_2_: 50 μm) operated with a motorized micromanipulator (Unisense A. S., Denmark). Oxygen profiles were measured separately at 50 μm resolution, while pH and H_2_S were conjointly recorded with a 200 μm resolution. The sensors were calibrated by following standard calibration procedures as described previously [17]. The measured values of H_2_S were recalculated as ΣH_2_S = [H_2_S] +[HS^−^] +[S^2−^] based on the recorded pH profile [30].

### 2.3. Laboratory sediment incubations

During each of the three campaigns, three intact sediment cores were incubated under anoxic conditions. To induce anoxia, cores were sealed using an air-tight polyoxymethylene lid equipped with two sampling ports made from gas-tight tygon tubing and placed inside a custom-made incubation chamber. The incubation temperature was the same in all campaigns (4°C, i.e. the *in situ* temperature of the bottom water in March). This allowed the comparison of fluxes and rates without the confounding temperature effect. The incubations lasted until H_2_S became detectable in the overlying water. The O_2_ concentrations were continuously recorded using Oxygen Spot Sensors (OXPSP5; Pyroscience, Germany) on the inside of the core liner. Additionally, the overlying water was discretely sampled on a weekly basis for Dissolved Inorganic Carbon (DIC), H_2_S, ammonium (NH_4_^+^), phosphate (PO_4_^3−^), dissolved iron (dFe) and dissolved manganese (Mn^2+^). Special care was taken to avoid oxygen intrusion during the sampling process. To collect water samples, glass syringes (Hamilton, USA) were connected to the sampling ports. After water collection, the overlying water was partially replaced with freshly prepared artificial seawater (Instant Ocean, salinity 28) that was flushed with N_2_ to remove O_2_. To this end, the lid was opened and approximately three-quarters of the overlying water were removed with a syringe. New anoxic water was then carefully poured onto a piece of bubble wrap placed over the sediment surface to prevent its disturbance. Subsequently, the air-tight lid was placed on the cores and the remaining air bubbles were removed via the syringes connected to the sampling ports. The replacement of the water typically took ~20 min.

### 2.4. Flux measurements

About 25 ml of overlying water was collected from each core incubation at each time point. For H_2_S analysis, 10 ml was immediately fixed with 1 ml ZnAc (5%) in a centrifuge tube (TTP, Switzerland) and stored at 4°C until analysis. The concentrations of H_2_S were determined according to the methylene blue method (limit of detection of ~3 µM) [31]. For DIC analysis, ~5 ml was withdrawn into a headspace vial and fixed with 5 µl of saturated HgCl_2_. Analysis was performed using an AS-C3 analyzer (Apollo SciTech, USA), consisting of an acidification and purging unit in combination with a LICOR-7000 CO_2_/H_2_O Gas Analyzer (precision 0.3%). For NH_4_^+^ and PO_4_^3−_,_^ 6 ml plastic vials were filled to the rim and stored at 4°C for <48 h before being analyzed using standard colourimetric methods on a SEAL QuAAtro segmented flow analyzer [32]. For dFe and dissolved manganese (dMn), 4 ml of the overlying water was fixed with 250 µl HNO_3_ (65%, suprapure, Merck, USA) and stored at 4°C until further analysis. Total concentrations of dissolved cations were subsequently determined using Inductively Coupled Plasma–Optical Emission Spectroscopy (ICAP 6600 ThermoFisher, USA).

To calculate the weekly efflux from the sediment $J_{S}$ for a given solute ‘S’, we calculated the difference of the solute inventory in the overlying water at the start and the end of the time interval (here ΔT = 7 days).


$$J_{S}=\frac{V_{OLW}\left({\left[S\right]}_{i,end}-{\left[S\right]}_{i,start}\right)}{\Delta T\;A_{core}},$$

where $V_{OLW}$ is the volume of overlying water on top of the sediment core and $A_{core}$ is the surface area of the sediment. The concentration ${\left[S\right]}_{i,end}$ is the measured concentration at the end of the *i*th time interval. The concentration at the start of the time interval can be written as ${\left[S\right]}_{i,start}=\alpha {\left[S\right]}_{medium}+\left(1-\alpha \right){\left[S\right]}_{i-1,end}$ where ${\left[S\right]}_{medium}$ is the solute concentration in the newly added medium (deoxygenated artificial seawater), α denotes the fraction of the total volume that is replaced (determined by measuring the height of the remaining water before adding new water) and ${\left[S\right]}_{i-1,end}$ is the solute concentration measured at the previous sampling point (*i* − 1). Mean fluxes are reported as mean ± standard deviation.

### 2.5. Pore water and solid-phase analyses

Down-core depth profiles were recorded for both the pore water and solid-phase geochemistry at the start (two replicate cores) and at the end (three replicate cores) of the incubations. To this end, sediment cores were transferred to an anaerobic glove box (Coy Lab Products, USA; N_2_ atmosphere with 3–5% H_2_). Each core was sectioned at 0.5 cm resolution up to 5 cm and then with a 1 cm resolution up to a maximum of 15 cm. Each sediment section was transferred into a 50 ml centrifuge tube (TTP, Switzerland), which was closed off in the glove box and transferred to an external centrifuge (3000 rpm, 10 min). After centrifugation, the tubes were transferred back into the glove box, the supernatant was filtered through a 0.45 µm cellulose filter (Millex-HA filter, Merck Millipore, USA) and distributed for different analyses (cations: 50 µl; NH_4_^+^: 200 µl; SO_4_^2−^: 250 µl; A_T_: 350 µl; H_2_S: 1 ml). The analysis and fixation of the pore water solutes were similar to those described for the overlying water. Note that, due to centrifugation, a fraction of H_2_S will degas, so reported concentrations should be considered a lower limit.

The A_T_ determination was based on the analysis of DIC after equilibration with an ambient atmosphere [33]. For SO_4_^2−^, 250 µl of the overlying water was fixed with 1 ml of a 10 mM ZnAc solution in a 15 ml centrifuge tube and stored at −20 °C. The concentration of SO_4_^2−^ was determined by ion chromatography using Na_2_CO_3_ (3.5 mM) and NaHCO_3_ (1.0 mM) buffer as the eluent on a Dionex AS14 analytical column (Thermo Scientific) equipped with a conductivity detector (ED40 electrochemical detector LC-02).

After centrifugation, the solid phase in 50 ml centrifuge tubes was retained after the removal of the supernatant. Air-tight aluminium bags (to protect samples from oxygen exposure during storage) were filled with the centrifuge tubes inside the anaerobic glove box, sealed and stored at −20°C until further analysis. Sedimentary Fe phases were determined using sequential extractions. The solid-phase iron oxide fraction reported here (FeO_x_, mostly ferrihydrite and lepidocrocite) was extracted for 24 h under continuously agitated conditions with 1 M hydroxylamine hydrochloride in 25% v/v acetic acid. Prior to the extraction, we removed the iron carbonate and FeS phases using a 1 M sodium acetate/acetic acid solution (pH 4.5, 24 h extraction under continuously agitated conditions). The extraction procedure essentially comprises the first two steps of the more elaborate extraction protocol presented in Poulton and Canfield [34]. Note that some ferrihydrite can be extracted during the first step and so FeO_x_ reported here provides a lower-bound estimate of the iron oxide fraction that is reactive with H_2_S [35].

## 3. Results

### 3.1. Field conditions upon core collection

**Figure 2.**
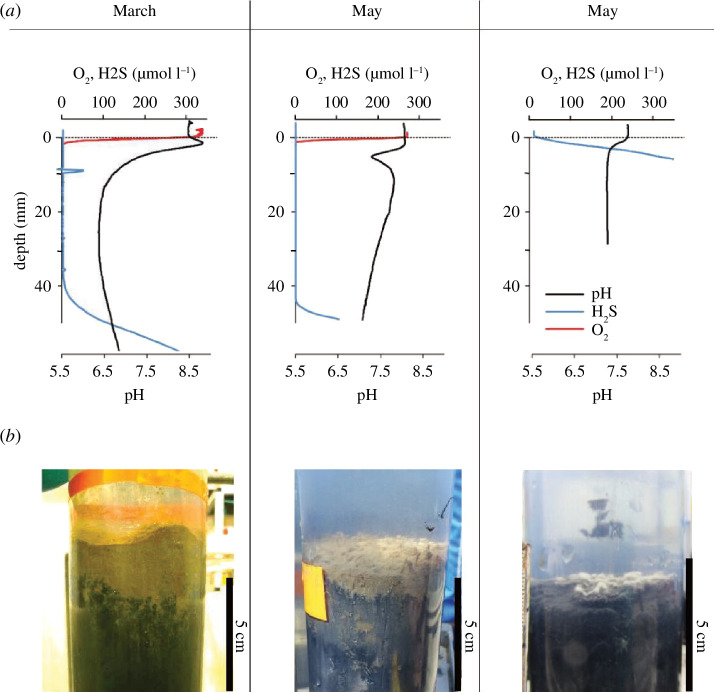
(*a*) Representative microsensor depth profiles of pH, H_2_S and O_2_ for one of the incubated sediment cores in March, May and August. (*b*) Images of the sediment cores after on-board retrieval.

The bottom water in 2015 followed the typical seasonal oxygenation trend: O_2_ levels are near air saturation in March (329 µmol l^−1^), undersaturated in May (175 µmol l^−1^) and anoxic in August (<1 µmol l^−1^; figure 1*c*). The sediment appearance and colouration showed marked differences between the three sampling times (figure 2*b*). In March, a large light-brown oxidized layer (thickness ~30 mm) was visible on the top of the darker black sediment. No signs of epifauna or burrow structures were apparent at the sediment surface. In May, this light-brown layer was reduced in size (~15 mm), followed by a light-grey sediment layer (~25 cm) before the dark black sediment started. Small polychaete tubes were sticking out of the sediment surface (~15 per core; approx. 5000 tubes m^−2^). In August, the sediment was completely black and the top layer appeared loose and flocculent (‘fluffy’).

The high-resolution depth profiles of pH, H_2_S and O_2_ confirmed the differences in sediment geochemistry among the three campaigns (figure 2*a*; underlying data in the electronic supplementary material [36]). In March and May, a wide zone devoid of O_2_ and ΣH_2_S was present in all sediment cores: 41 ± 4 mm in March and 39 ± 5 mm in May. In August, ΣH_2_S increased steeply right below the sediment–water interface. In March, the pH depth profile exhibited a pH maximum in the oxic zone (pH > 8.5), typically for cable bacteria and a pH minimum (pH < 6.5) at the base of the suboxic zone, while in May, a distinct pH minimum (pH ~ 7) occurred at the base of the oxic zone and in August, the pH decreased slightly in the top millimetres to attain a stable pH (pH ~ 7) at depth.

### 3.2. Sediment geochemistry

**Figure 3.**
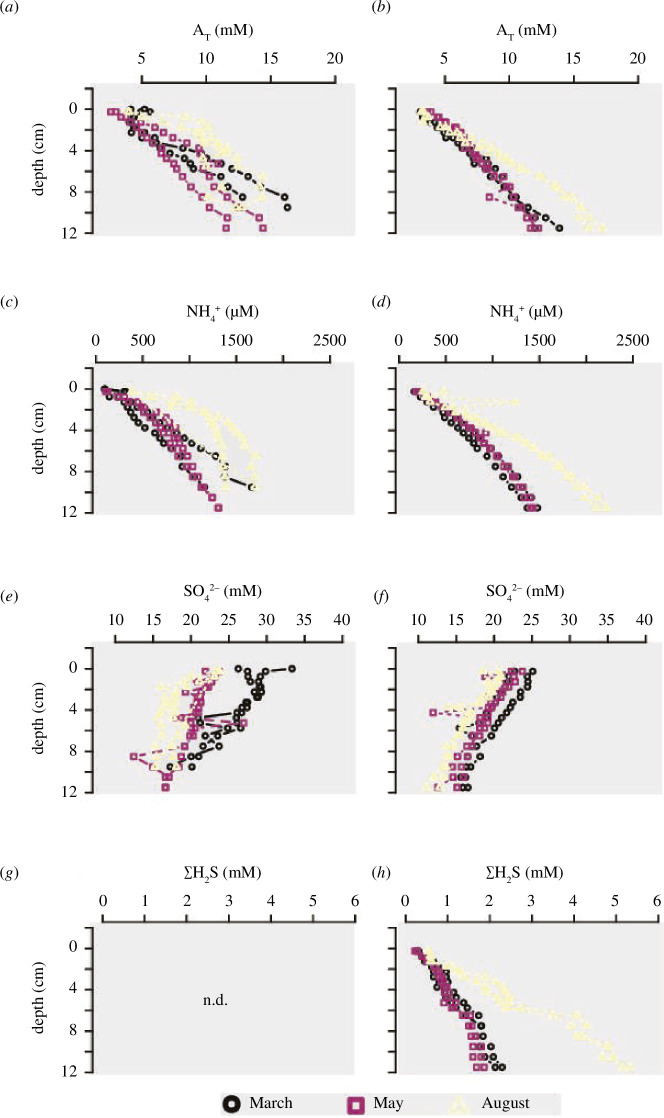
Pore water depth profiles of (*a*,*b*) total alkalinity (A_T_), (*c*,*d*) NH_4_^+^, (*e*,*f*) sulfate (SO_4_^2−^) and (*g*,*h*) ∑H_2_S at the start (left) and end of the incubation (right). Pore water ∑H_2_S was not measured before incubation.

**Figure 4.**
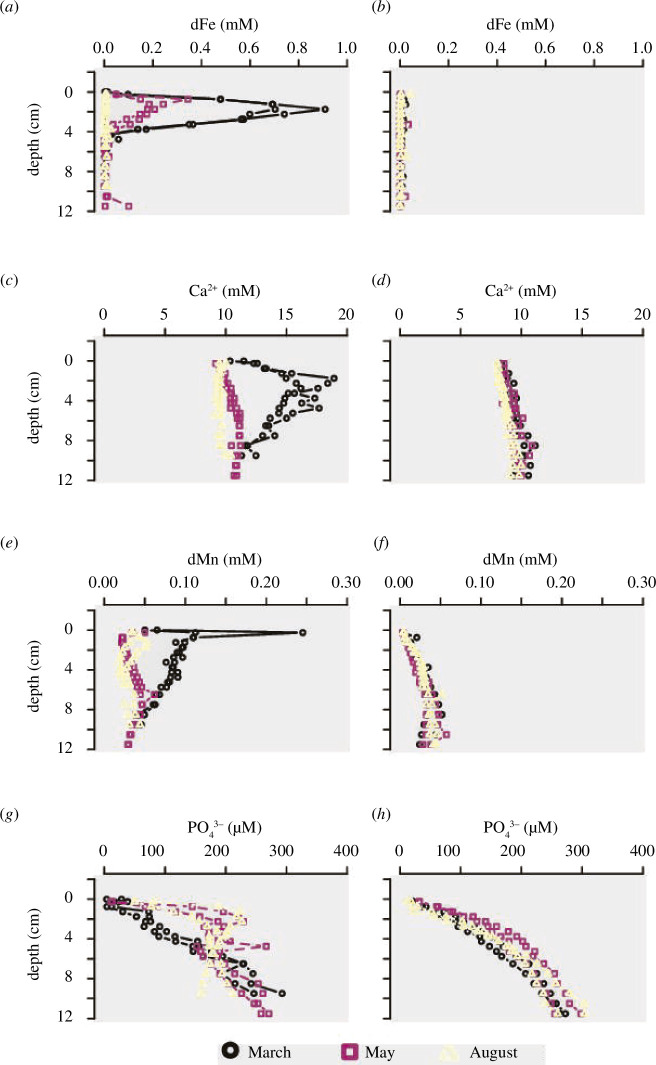
Pore water depth profiles of (*a*, *b*) dFe, (*c, d*) calcium (Ca^2+^), (*e, f*) dMn and (*g*, *h*) PO_4_^3−^ at the start (left) and end of incubation (right).

**Figure 5.**
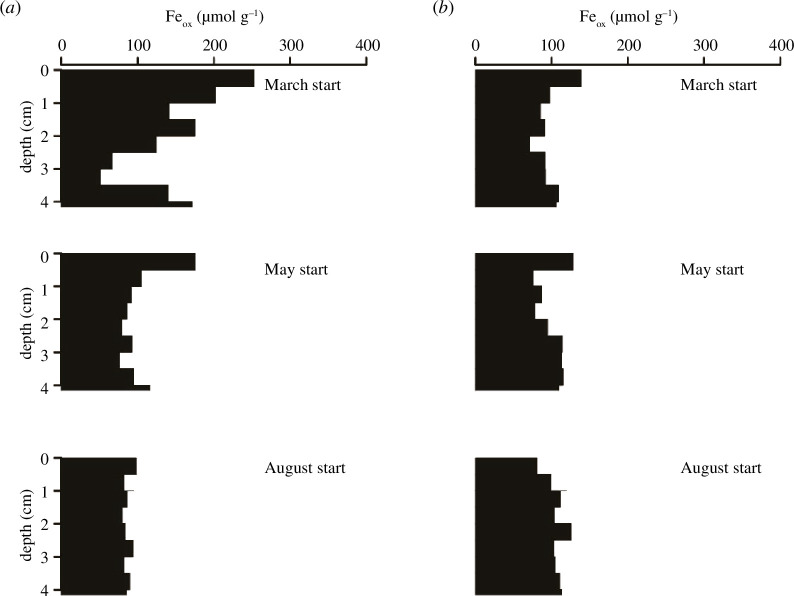
Depth profiles for FeO_x_ before (*a*) and after (*b*) anoxic incubation.

Detailed pore water and solid-phase analyses were performed before and after anoxic incubations (figures 3–5; underlying data in the electronic supplementary material [36]). After incubation, all cores showed similarly shaped pore water profiles. Mineralization products (NH_4_^+^, PO_4_^3−^, A_T_ and H_2_S) showed a gradual increase from the sediment–water interface downwards and inversely, SO_4_^2−^ showed a gradual decrease in deeper layers (figures 3 and 4*g*,*h*). No substantial amounts of dFe were detected in the pore water (figure 4*a*,*b*), while Ca^2+^ and dMn showed a slight and steady increase with depth (figure 4*c*–*f*).

The starting conditions before incubation mirror the distinct geochemical settings between time points. Notably, the depth profiles of dFe, dMn and Ca^2+^ (figure 4*a*–*f*) differed strongly among the three seasons. A large mobilization of dFe was visible in the first 4 cm in March (up to 0.9 mmol l^−1^), some dFe mobilization was still apparent in May (up to 0.2 mmol l^−1^), while only traces of dFe (maximum 5 µmol l^−1^) were present in August. In March, Ca^2+^ accumulated between 1 and 9 cm depth, while in May and August, the pore water Ca^2+^ concentrations remained constant at the level of the overlying water (~10 mmol l^−1^). dMn showed a seasonal pattern comparable to Ca^2+^, with high accumulation in the first few centimetres in March. The end products of organic matter mineralization (NH_4_^+^, A_T_ and SO_4_^2−^) reflected the seasonal effect of temperature on SO_4_^2−^ reduction (figure 3*a*–*f*). NH_4_^+^ and A_T_ increased more steeply and SO_4_^2−^ decreased more steeply in August, indicating higher SO_4_^2−^ reduction rates compared to March and May. In March, a subsurface A_T_ minimum indicated alkalinity consumption in the suboxic zone, while a subsurface SO_4_^2−^ maximum indicated substantial H_2_S oxidation. In May or August, these subsurface A_T_ minima and SO_4_^2−^ maxima were not present. PO_4_^3−^ pore water profiles showed a similar trend among all cores before and after incubation (figure 4*g*–*h*). PO_4_^3−^ concentrations increased quickly in the first centimetres (start: first 3 cm to 200 µmol l^−1^; end: first 5 cm to 220 µmol l^−1^), below which the increase slowed down (start: ~220 µmol l^−1^; end: 250 µmol l^−1^). The starting conditions for March were however notably different: after the initial rise in PO_4_^3−^ in the first 1.5 cm (from 0 to 70 µmol l^−1^), a depletion of PO_4_^3−^ was observed between 2 and 7 cm deep.

The inventories of solid-phase iron (figure 5; underlying data in the electronic supplementary material [36]) showed an enrichment in the FeO_x_ pool in the top layer in March (up to 152 µmol g^−1^ in first 1 cm) and May (up to 70 µmol g^−1^ in first 0.5 cm). At the end of anoxic incubations, FeO_x_ decreased in both the March and May cores. In contrast, in August, FeO_x_ inventories remained similar before and after incubation.

### 3.3. Fluxes across the sediment–water interface

**Figure 6.**
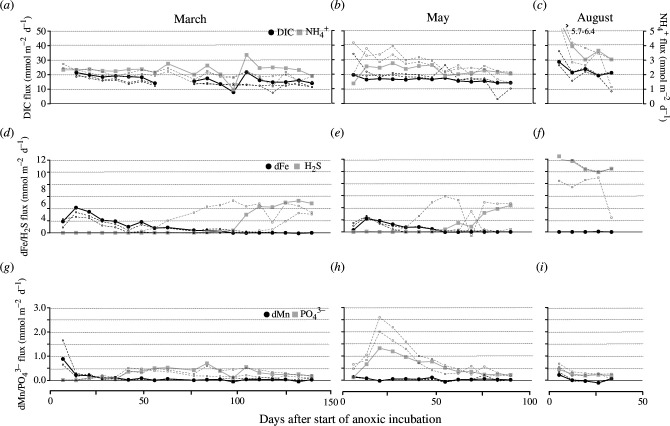
Fluxes of (*a*–*c*) DIC and NH_4_^+^, (*d*–*f*) ∑H_2_S and dFe and (*g*–*i*) PO_4_^3−^ and dMn in the three sets of incubations. The filled symbols and lines are the first replicate core and the unfilled symbols and dashed lines show the other two replicates. Note that the scale for NH_4_^+^ in panels a–c is on the right and the range of off-scale values in panel c are indicated by the arrow.

The fluxes of DIC, NH_4_^+^, dFe, PO_4_^3−^, ΣH_2_S and dMn between the sediment and the overlying water were determined at weekly resolution (figure 6; underlying data in the electronic supplementary material [36]). The mineralization end products of organic matter (DIC and NH_4_^+^) showed always an efflux out of the sediment. In the March and May cores, the fluxes of DIC and NH_4_^+^ co-varied and were approximately constant throughout the incubation period. The mean effluxes of DIC (14 ± 3 mmol C m^−2^ d^−1^) and NH_4_^+^ (2.0 ± 0.3 mmol N m^−2^ d^−1^) in the March cores were slightly lower than those in the May cores (17 ± 2 mmol C m^−2^ d^−1^ and 2.5 ± 0.5 mmol N m^−2^ d^−1^), but gave rise to a similar C:N ratio of 7.1 ± 0.9. In August, the *in situ* temperature of the bottom water (18°C) was substantially higher than the incubation temperature of all campaigns (4°C). This likely led to an acclimation effect at the start of the incubation: the DIC efflux started high (30 mmol m^−2^ d^−1^) before stabilizing around 20 mmol m^−2^ d^−1^. Similarly, the NH_4_^+^ efflux also started high (5.2 mmol m^−2^ d^−1^) before decreasing to 2.2 mmol m^−2^ d^−1^, providing a mean C:N ratio of 6.1 ± 1.1 (figure 6*a*–*c*).

The time point at which a detectable ΣH_2_S efflux was first observed differed substantially between sampling campaigns (figure 6*d*–*f*). In the August cores, we observed a ΣH_2_S efflux from the start of the incubation (ΣH_2_S efflux 10–12 mmol m^−2^ d^−1^). In contrast, in the March and May cores, there was a long initial period with no detectable ΣH_2_S efflux. In the March cores, it took 103 ± 25 days before H_2_S was released from the sediment, while in the May cores, the ΣH_2_S efflux became detectable after 56 ± 15 days. In both cases, when H_2_S appeared in the overlying water, the ΣH_2_S efflux quickly increased over a period of 3–4 weeks, after which it stabilized at a constant value. In the March cores, this steady-state ΣH_2_S efflux was 3.7 ± 1.1 mmol S m^−2^ d^−1^ and in the May cores, it attained a similar value (4.1 ± 1.2 mmol S m^−2^ d^−1^). The C:S ratio of steady-state effluxes was 2.3 ± 0.5 in the August cores—close to the theoretical C:S ratio of 2 for SO_4_^2−^ reduction—but amounted to 3.8 ± 1.2 in the March cores and 4.1 ± 1.3 in the May cores, suggesting some form of reduced S retention in the sediment and/or DIC produced by carbonate dissolution.

All cores (*n* = 3) from the March campaign showed a similar pattern in dMn, dFe and ΣH_2_S fluxes (figure 6*d*–*i*). First, an efflux of dMn was observed, which initially increased (up to 0.9 mmol Mn m^−2^ d^−1^) and then decreased (the total period of dMn release lasted <2 weeks). Subsequently, dFe is released from the sediment and the efflux increased to a maximum in the second week (2.5–4 mmol Fe m^−2^ d^−1^) before dropping gradually to zero (between days 50 and 105). ΣH_2_S started to release from the sediment immediately after the last detectable dFe efflux was measured. The sequential release pattern in the May cores was similar to that in the March cores but occurred over a shorter period of time (figure 6*e,h*). In the May cores, the initial dMn efflux was also slightly lower (up to 0.10 mmol Mn m^−2^ d^−1^) than for the cores collected in the March campaign and the dFe efflux also peaked in the second week (up to 2.7 mmol dFe m^−2^ d^−1^), after which it slowly decreased. As in the March cores, a detectable ΣH_2_S efflux was only observed in the week after the last detectable efflux of dFe.

In the March cores, phosphate (PO_4_^3−^) fluxes were not detectable over the two first weeks of the incubation experiment (figure 6*g*). After four weeks, the PO_4_^3−^ effluxes rapidly increased from 0.05 to 0.45 mmol m^−2^ d^−1^ over the course of one week, followed by a more gradual increase up to 0.7 mmol m^−2^ d^−1^ until day 98. When the efflux of ΣH_2_S started, PO_4_^3−^ effluxes decreased to 0.2 mmol m^−2^ d^−1^. In the May cores, they started immediately and increased to 1.1 mmol m^−2^ d^−1^ on day 21. Afterwards, they decreased to 0.4 mmol m^−2^ d^−1^ at the end of incubation. In the August cores, PO_4_^3−^ fluxes stayed at a comparable level throughout the incubation (0.1–0.4 mmol m^−2^ d^−1^).

## 4. Discussion

### 4.1. Seasonality in sedimentary biogeochemical cycling in Lake Grevelingen

**Figure 7.**
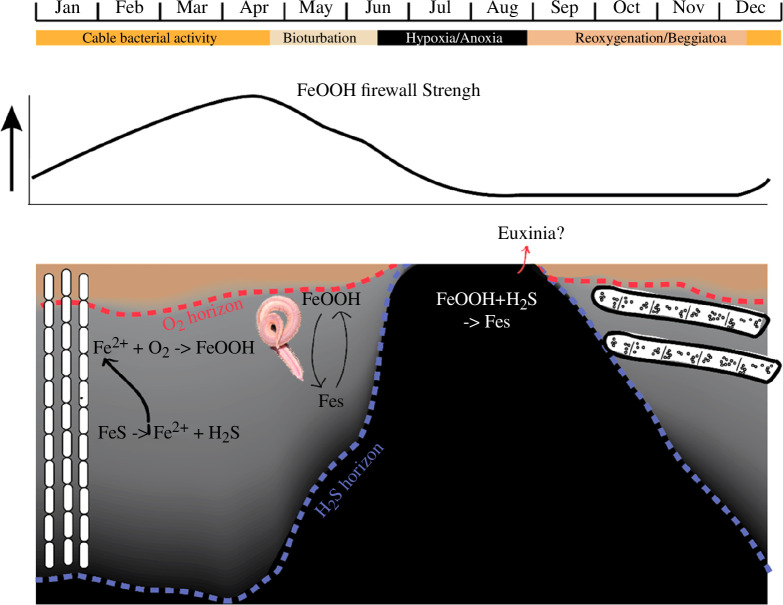
Schematic of the seasonal cycle in the seasonal hypoxic Lake Grevelingen. In winter/early spring, cable bacteria promote the build-up of FeOOH in the oxic zone and deplete FeS in the suboxic zone. Afterwards, bioturbation-induced mixing leads to down-mixing of FeOOH (which is transformed in FeS). In summer, hypoxia/anoxia occurs and H_2_S will further deplete FeOOH. Finally, after the first reoxygenation, Beggiatoa recolonizes the sediment.

The seasonal depletion of oxygen in the bottom water imposes a pronounced seasonality on the population dynamics of the sediment infauna and microbial communities which profoundly affect the sedimentary geochemical cycling of sulfur in Lake Grevelingen. Seitaj *et al*. [9,19,25] proposed a model for the seasonal iron and sulfur cycling in the sediments of Lake Grevelingen, which distinguishes four consecutive biogeochemical regimes throughout the seasonal cycle: (i) electrogenic sulfur oxidation by cable bacteria occurs from winter to spring, (ii) bioturbation-induced metal cycling becomes prominent in late spring and early summer, (iii) anoxic conditions dominate throughout summer (figure 7) and (iv) sulfur oxidation by *Beggiatoaceae* rises in fall right after bottom water ventilation.

Overall, the dataset collected here fully aligns with the seasonal Fe and S cycling model reported by Seitaj *et al*. [9]. In March, the pore water chemistry revealed a clear imprint of electrogenic sulfur oxidation by cable bacteria (figure 2) [12–14], as indicated by alkaline pH peaks in the oxic zone and acidic pore waters in the suboxic zone (figure 2) and the associated dissolution of FeS (as indicated by strong dFe accumulation; figure 4*a*) and CaCO_3_ (as indicated by strong Ca^2+^ accumulation; figure 4*c*). Overall, the metabolic activity of cable bacteria drives the dissolution of FeS and stimulates the subsequent reoxidation of dFe to FeO_x_ in the oxic zone [15], which accumulates near the sediment surface (figure 5). In contrast, in May, the pore water chemistry showed the features of bioturbation-driven iron cycling, as indicated by the acidic pH minimum (figure 2) at the base of the oxic zone indicating iron re-oxidation [9,37], recovery to higher pH values below (indicative of iron reduction [37]; figure 2) and less pronounced FeO_x_ in the surface sediment (figure 5). Note that the alignment between the O_2_ decrease and the pH minimum is not perfect in May, which is likely caused by the uneven surface of the cores (see the core picture in figure 2). Since we set the surface for each individual profile and profiles of O_2_ and pH are taken at separate locations, it is not surprising to have mismatches in the depth between individual microprofiles. In August, no oxygen was present in the overlying water (figure 2) suggesting that the sediment geochemistry is governed by anoxic biogeochemical processes. Mineralization is dominated by sulfate reduction, while FeO_x_ is reduced back to FeS using the available H_2_S. The pH profile stays constant with depth, as is expected for sediment dominated by sulfate reduction and without significant iron cycling [9]. As a result, the stock of FeO_x_ is depleted to its background value throughout the depth in the solid phase of the sediment (figure 5). At the end of the incubation, all measured parameters showed near-identical down-core profiles, demonstrating that cores incubated in different seasons eventually all converged to the same type of geochemical cycling, i.e. organic matter mineralization dominated by sulfate reduction (figures 3 and 4) in combination with a small amount of calcium carbonate dissolution.

### 4.2. Strength of the microbial-induced firewall

The so-called ‘iron firewall’ hypothesis predicts that the build-up of FeO_x_ as a consequence of the metabolic activity of cable bacteria in spring in seasonally hypoxic systems imposes an oxidative barrier for sulfide, which hence prevents the efflux of ΣH_2_S when the oxygen in the bottom water becomes depleted in summer. Our experiments support the firewall hypothesis: sulfide fluxes are substantially delayed in the March cores (103 ± 25 days) when a large FeO_x_ stock is present in the top layer of the sediment (figure 5), while sulfide release is immediate in the cores sampled in late August when this FeO_x_ stock is depleted. The FeO_x_ stock in the sediment surface can originate from two processes; (i) external, via delivery of FeO_x_ from the water column through sedimentation and (ii) internal, via upward diffusion of reduced ferrous iron and subsequent reoxidation in the oxic zone [20,21]. The external source of FeO_x_ can be estimated from the surface concentration of FeO_x_ in August (the season when there is no active iron cycling in the sediment [9,19]) ([FeO_x_] = 50 µmol g^−1^) and the sediment accumulation rate (1.6 g cm^−2^ yr^−1^ [17]), which gives us an annually averaged external delivery of FeO_x_ of 2.1 mmol m^−2^ d^−1^. The internal source, which is the part of the FeO_x_ layer due to cable bacterial activity, equals the upward diffusing ferrous iron flux (1.2–1.5 mmol m^−2^ d^−1^). Hence, the supply of FeO_x_ to the surface sediment is 57–71% higher due to the activity of cable bacteria.

The recorded iron fluxes support the idea that FeO_x_ accumulated in the top layer prevents the efflux of sulfide. In all incubations, the start of the H_2_S efflux coincides with the end of the dFe flux. The reaction of FeO_x_ with free sulfide (sulfide-mediated iron dissolution) is a chemical process, where Fe^2+^ is formed (note that we write this two-step process as one reaction for simplicity; in reality, elemental sulfur is formed as an intermediate) [38–40].


$$HS^{-}+8FeOOH+15H^{+}\to SO_{4}^{2-}+8Fe^{2+}+12H_{2}O.$$

Subsequently, Fe^2+^ reacts with H_2_S to form FeS.


$$Fe^{2+}+HS^{-}\to FeS+H^{+}.$$

During the first step, Fe^2+^ is released into the pore water and as a consequence, part of the Fe^2+^ released can diffuse out of the sediment, rather than being trapped as FeS. This explains the observed efflux of Fe^2+^ out of the sediment. Note that the oxidation of organic matter can also reduce iron, yet sulfide-mediated iron dissolution is likely the dominant iron-reducing process in the non-bioturbated sediments investigated here [41,42]. dMn fluxes peak just before dFe fluxes (figure 5), which suggests that, as long as there are manganese oxides (MnO_2_) present, a fraction of the produced Fe^2+^ is initially reoxidized to FeOOH by MnO_2_ reduction [19].


$$2Fe^{2+}+MnO_{2}+2H_{2}O\to 2FeOOH+Mn^{2+}+2H^{+}.$$

At the field site, manganese oxides are present in concentrations that are <5% of the iron oxide concentrations [19], which is indicated by low dMn fluxes (4 times lower than dFe) and the rapid decrease to quasi-zero (figure 5). Hence, the impact of manganese on the eventual sulfide delay can be considered minor compared to the iron oxide firewall.

How strong is the firewall induced by the cable bacteria? In the March cores, we observe an inventory change of ~850 mmol m^−2^ of FeO_x_ between the start and the end of incubation (change in top 1 cm, figure 5). During incubation, the cumulative flux of dFe out of the sediment is 100 mmol m^−2^. If we suppose that dFe is only released from iron oxides, 750 mmol m^−2^ of dFe from FeO_x_ must be captured as FeS in the sediment (i.e. a trapping efficiency of 750/850 * 100 = 88%). Carbon mineralization is ~15 mmol m^−2^ d^−1^ in the cores over the course of incubation (as derived from the mean DIC flux a–c). Note that the DIC efflux is not solely the effect of organic mineralization, but can also increase due to carbonate dissolution. As a result, we can consider the DIC effluxes as an upper bound on the mineralization rate. Since anoxic carbon mineralization in Lake Grevelingen is dominated by sulfate reduction [9], one expects sulfate reduction rates in the range of ~7.5 mmol m^−2^ d^−1^ (based on the estimated organic matter mineralization rate and a stoichiometric S:C ratio of 1:2). Assuming that the total FeO_x_ inventory change was caused by sulfide-mediated iron dissolution, the consumption of free sulfide by FeO_x_ reduction amounts to (1/8) * 850 = 106 mmol S m^−2^, while the ensuing FeS precipitation removes another 750 mmol S m^−2^. Therefore, the accumulated FeO_x_ would be able to delay the sulfide release for at least 856 mmol m^−2^/ 7.5 mmol m^−2^ d^−1^ = 114 days in March. This estimate is highly congruent with the euxinia delay of 103 ± 25 days as observed in the incubation experiment. A similar sulfur budget calculation can be made for May incubation. This provides a theoretical delay of the sulfide release of 45 days, which is again comparable to the flux results from the incubations (the observed delay of the free sulfide release in the three incubated cores ranged between 40 and 60 days). Note that *in situ* temperatures during summer become higher and sulfide production would increase accordingly. The delay in sulfide release would thus be shorter than in our incubated cores.

In May, we observed small polychaetes at the sediment surface, consistent with the previous observations of sediment recolonization by juvenile macrofauna in late spring [9]. We hence contend that macrofaunal activity could induce the down-mixing of FeO_x_ through bioturbation [43,44]. A fraction of FeO_x_ in the top layer will be mixed down which will be reduced in deeper layers, thus accelerating the conversion of FeO_x_ into FeS (figure 7) and hence partially weakening the strength of the iron firewall. In August 2015, the iron firewall appeared to be completely exhausted and a sulfide efflux was detectable from the first week in the incubations (figure 6*f*). Because H_2_S was not detectable in the bottom water, it appears that our August sampling occurred at a moment when the iron firewall was exhausted by previous weeks of anoxia, but the bottom water did not have the chance yet to accumulate H_2_S in large concentrations. Alternatively, more turbulence created by a stochastic event (e.g. by strong winds) prior to our sampling in August could have led to the transient ventilation of the bottom water [45]. In either case, the bottom water of Lake Grevelingen was on the brink of developing euxinia. If we take the conservative estimate that the bottom 10 m of the water column is well-mixed and adopt a flux of 12 mmol m^−2^ d^−1^ of H_2_S (as measured in August, figure 6*f*), about 17 days of efflux are needed to reach 20 µmol l^−1^ H_2_S, a threshold above which eukaryotic mitochondria become poisoned [7]. Therefore, an increase in the hypoxia length of a few weeks as a result of climate change (e.g. caused by an earlier onset of stratification in spring and/or increased bottom waters temperatures and higher mineralization rates in summer) would hence increase the risk of developing euxinia in Lake Grevelingen.

### 4.3. Effect on phosphorus cycling

Phosphorus (P) is an essential nutrient (in combination with nitrogen) for primary production in coastal systems and is intimately linked to the iron cycle [23]. In Lake Grevelingen, the formation of FeO_x_, stimulated by electrogenic sulfur oxidation, was proposed to prevent the efflux of P from the sediment during spring, while the dissolution of the FeO_x_ layer during summer led to a higher release of P from the sediment [25]. The flux pattern in our incubations fully aligns with this model proposed by Sulu-Gambari *et al*. [25]. In March, the PO_4_^3−^ effluxes only started 14 days after the start of the incubation and reached a maximum of 0.9 mmol m^−2^ d^−1^. In May, the fluxes of P immediately started and reached a much higher rate in a shorter period of time. In spring, the newly formed FeO_x_ layer had a large capacity to bind phosphorus, but due to the cable bacterial activity and the formation of FeO_x_, the binding capacity for P was not yet fully exhausted. This could explain the two-week lag in P effluxes in March. Later in the season, when cable bacterial activity had ceased and the formation of the FeO_x_ had stopped, the FeO_x_ pool likely became saturated with PO_4_^3−^, explaining why P effluxes are higher in May compared to March and why they immediately start right after the induction of anoxia. In Lake Grevelingen, the benthic–pelagic coupling of P is consequently heavily regulated by the activity of the cable bacteria ([25]; this study). In spring, a large pool of FeOx was formed, which can keep most P in the sediment or even promote the capture of additional P into the sediment. As such, the presence of cable bacteria can induce a large retention of P within the sediment which leads to amplified P efflux once hypoxia sets in.

### 4.4. Outlook: cable bacteria as ecosystem engineers

The metabolic activity of cable bacteria appears to have a large impact on the biogeochemical cycling of Fe, Mn, P, S and trace elements in Lake Grevelingen [9,19,25,46,47]. The build-up of FeO_x_ in the spring is an immediate consequence of the acidifying metabolism of cable bacteria and our experiments demonstrate that this large FeO_x_ pool forms an effective barrier against sulfide release from the sediment later in the hypoxia season. Moreover, this FeO_x_ pool efficiently retains P in the sediment. As a result, cable bacteria can be thought of as microbial ecosystem engineers. Given the toxicity of sulfide for organisms and the large detrimental impact of sulfide on coastal ecosystems, the capability of delaying or even preventing euxinia emerges as a major structuring factor in coastal ecosystems. The occurrence of cable bacteria in other seasonal hypoxic environments [16,27,28] hints towards a similar function and thus suggests that the iron firewall mechanism could be more widespread. Moreover, in order to determine the future prevalence of euxinia, it is appropriate to investigate how cable bacteria and their iron firewall mechanism will respond to a warming coastal ocean. In 2015, the strength of the iron firewall appeared to match the length of the hypoxia regime in Lake Grevelingen, preventing the development of euxinia in late summer. Still, the system appears right on the brink of developing euxinia and in the near future, our results suggest that H_2_S-rich bottom waters may form in warmer years with prolonged stratification periods.

## Data Availability

All data are included in the supplementary materials [36].
